# Novel Application of a Multiscale Entropy Index as a Sensitive Tool for Detecting Subtle Vascular Abnormalities in the Aged and Diabetic

**DOI:** 10.1155/2013/645702

**Published:** 2013-02-20

**Authors:** Hsien-Tsai Wu, Men-Tzung Lo, Guan-Hong Chen, Cheuk-Kwan Sun, Jian-Jung Chen

**Affiliations:** ^1^Department of Electrical Engineering, National Dong Hwa University, Hualien, No. 1, Section 2, Da-Hsueh Road, Shoufeng, Hualien 97401, Taiwan; ^2^Research Center for Adaptive Data Analysis and Center for Dynamical Biomarkers and Translational Medicine, National Central University, Chungli, Taiwan; ^3^Department of Emergency Medicine, E-Da Hospital, I-Shou University, Kaohsiung City, Taiwan; ^4^Department of Chinese Medicine, Buddhist Tzu-Chi General Hospital Taichung Branch, Taichung, Taiwan; ^5^School of Chinese Medicine, Tzu Chi University, Hualien, Taiwan

## Abstract

Although previous studies have shown the successful use of pressure-induced reactive hyperemia as a tool for the assessment of endothelial function, its sensitivity remains questionable. This study aims to investigate the feasibility and sensitivity of a novel multiscale entropy index (MEI) in detecting subtle vascular abnormalities in healthy and diabetic subjects. Basic anthropometric and hemodynamic parameters, serum lipid profiles, and glycosylated hemoglobin levels were recorded. Arterial pulse wave signals were acquired from the wrist with an air pressure sensing system (APSS), followed by MEI and dilatation index (DI) analyses. MEI succeeded in detecting significant differences among the four groups of subjects: healthy young individuals, healthy middle-aged or elderly individuals, well-controlled diabetic individuals, and poorly controlled diabetic individuals. A reduction in multiscale entropy reflected age- and diabetes-related vascular changes and may serve as a more sensitive indicator of subtle vascular abnormalities compared with DI in the setting of diabetes.

## 1. Introduction

Endothelial dysfunction (ED) has been documented as a sign of the imminent onset of cardiovascular disease (CVD) including atherosclerosis and CVD-related disorders (i.e., diabetes, hypertension) [1–3]. The commonly used noninvasive means of assessing ED include flow-mediated dilatation (FMD) [4–6] and reactive hyperemia peripheral arterial tonometry (RH-PAT) [7–9]. The principle underlying the measurement is the induction of transient ischemia through increased cuff pressure over the upper arm, followed by a release of pressure. The reperfusion thus produced elicits reactive hyperemia (RH) in the distal blood vessels through the release of nitric oxide (NO), which is an indicator of endothelial integrity [10–12].

Although FMD provides direct information about the changes in blood vessel diameter, it requires an experienced operator and expensive equipment. On the other hand, RH-PAT acquires arterial pulse signals of the index finger through tonometry and compares them before and after RH induction. The popularity of its clinical use, however, is also hampered by the need for experienced personnel and its costly disposable accessories. As a result, no well-designed study has investigated ED in elderly and diabetic subjects who are at high risk of CVD. The importance of early detection of ED in diabetic patients is further underscored by the finding that ED occurs within 10 years of full-fledged diabetes. Early detection of ED and timely intervention, therefore, are of utmost importance in the prevention of diabetes and its associated complications [13–15].

This study was designed to test the sensitivity and validity of applying a novel multiscale entropy index (MEI) in evaluating the degree of ED in subjects at risk of CVD. This was performed by analyzing the dynamical complexity of arterial pulse waveform signals, obtained through the wrist before and after induction of RH from 4 different subject populations using multiscale entropy analysis of biological signals [16–19].

## 2. Materials and Methods

### 2.1. Study Population and Grouping

A total of 70 subjects were recruited from the diabetes outpatient clinic of Hualin Hospital between December 2009 and October 2010. In addition, there were 70 healthy controls recruited from a health examination program at the same hospital. The 140 study subjects were categorized into the following 4 groups: group 1, which included healthy young individuals aged 20–30 years, with no known history of CVD, glycosylated hemoglobin (HbA1c) levels of less than 6%, and fasting blood sugar levels of less than 126 mg/dL; group 2, which included healthy middle-aged or elderly individuals aged 40–70 years, with no known history of CVD, HbA1c levels of less than 6%, fasting blood sugar levels of less than 126 mg/dL, and absence of metabolic syndrome according to the ATP III report [20]; group 3, which included well-controlled diabetic individuals aged 50–80 years, with an established diagnosis of type 2 diabetes (i.e., HbA1c levels >6.5% and fasting sugar levels >126 mg/dL) [20], HbA1c levels between 6.5% and 8% and fasting blood sugar levels of more than 126 mg/dL at the time of the present study; group 4, which included poorly controlled diabetic individuals aged 50–80 years, who fit the criteria of diabetes with HbA1c levels of more than 8% and fasting sugar levels of more than 126 mg/dL.

### 2.2. Experimental Procedure

Before initiating the study, subjects were required to fill out a questionnaire on basic demographic and anthropometric data as well as information on lifestyle and personal/family history of CVD. Physicians also obtained blood samples after 8 hours of fasting for determination of serum high-density lipoprotein (HDL), low-density lipoprotein (LDL), triglyceride (TG), fasting blood sugar, and HbA1c levels. Informed consent was obtained from all subjects.

The study subjects were allowed to assume a supine position and rest in a quiet, temperature-controlled (25°C) room for 5 minutes before measurement. Blood pressure was obtained once over the left arm of the supine patients using an automated oscillometric device (BP3AG1, Microlife, Taiwan) with a cuff of appropriate size. One pressure cuff of the air pressure sensing system (APSS) was then put around the left arm, whereas the other cuff was applied on the left wrist [21, 22]. The pressure of the cuff around the wrist was maintained at 40 mmHg throughout the process of measurement, which took 17 minutes for each subject.

### 2.3. Dilatation Index (DI) Computation

**Figure 1:**
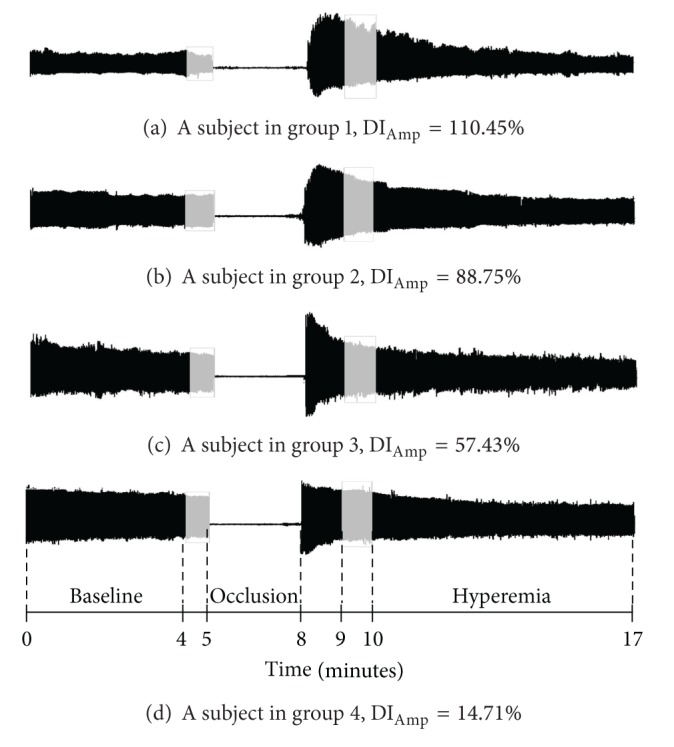
Representative arterial pulse signals from the 4 different groups, showing variations in the dilatation index (DI_Amp_). Group 1: healthy young individuals; group 2: healthy middle-aged or elderly individuals; group 3: well-controlled diabetic individuals; group 4: poorly controlled diabetic individuals.

The structure and principles of operation of the APSS have been previously reported [21]. In brief, the APSS system consists of two sets of pressure cuffs, a piezoresistive sensor, and an endothelial function measurement module board. The first set of pressure cuffs is placed over the upper arm and triggers the endothelial function, whereas the second set is placed over the wrist for data acquisition. The piezoresistive sensor, which is connected to the second set of pressure cuffs, is used to detect the pulse wave and record the arterial waveform in the system. The endothelial function measurement module board amplifies and filters the captured arterial waveform. The pressure detected by the piezoresistive sensor was thus converted into electrical signals which were then amplified and filtered to obtain the analog signals. The analog signals were digitized with an analog-to-digital converter (Model MSP430F449, Texas Instruments, TX, USA) at a sampling rate of 500 Hz and stored in a computer for later analysis [22]. The total duration of signal acquisition was 17 minutes (Figure 1), which consisted of 5 minutes of data recording at a wrist cuff pressure of 40 mmHg with the arm cuff deflated (i.e., the baseline), 3 minutes of blood flow occlusion by increasing the cuff pressure of the upper arm to 200 mmHg (i.e., the occlusion phase), and 9 minutes of data acquisition after complete deflation of the pressure cuff over the upper arm with the pressure of the wrist cuff being maintained at 40 mmHg throughout (i.e., the hyperemic phase). The amplitude of the signals during the hyperemic phase varied with the subject's age and disease status (see Figure 1). The mean amplitude of signals within a representative one-minute period between the fifth and tenth minute after the beginning of data collection was selected from the baseline and hyperemic phases, respectively, and labeled as Amp_Baseline_ and Amp_RH_ (see Figure 1). The dilatation index (DI) [8, 21] of the forearm blood vessel is defined as

(1) $$\begin{array}{c} \text{D}{\text{I}}_{\text{Amp}}=\frac{{\text{Amp}}_{\text{RH}}}{{\text{Amp}}_{\text{Baseline}}}\times 100\%. \end{array}$$

In agreement with the results of previous studies [8, 9, 22], our finding (see Figure 1) showed that the value of DI decreases with advancing age and increasing severity of diabetes. In contrast with the calculation of DI, which adopted 1 minute of signals from both the baseline and reactive hyperemic phases, the present study attempted to utilize the entire 14 minutes of signals (except for the occlusion phase) in the calculation of the multiscale entropy index (MEI). This was performed to provide a sensitive tool for detecting subtle vascular abnormalities in the elderly and diabetic patients.

### 2.4. Multiscale Entropy Index (MEI)

**Figure 2:**
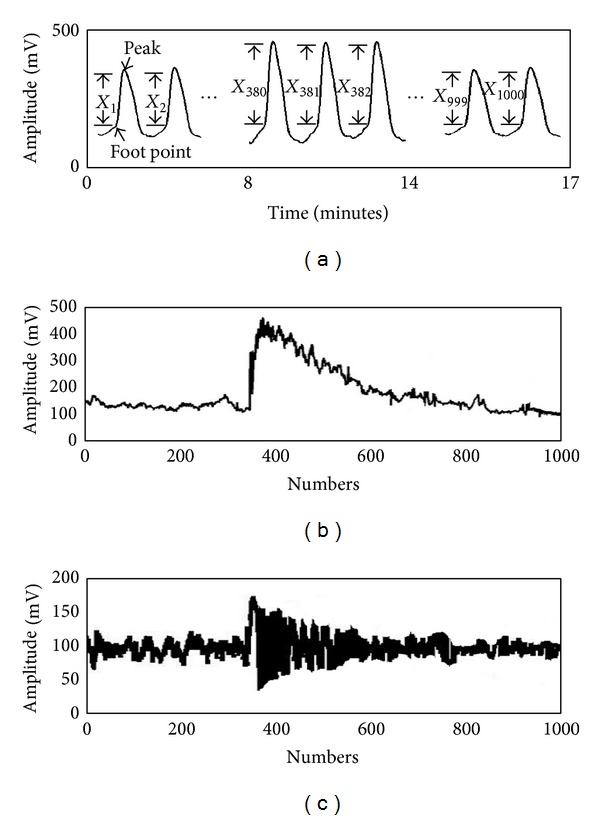
(a) Identification of the footpoint and peak of each arterial waveform measured from the wrist of a healthy young subject (group 1) using the air pressure sensing system (APSS), after connecting the baseline signals (5 min) to those at the hyperemic phase (9 min). (b) Plotting of the amplitudes from 1000 waveforms against time, giving a nonstationary curve. (c) Final curve after detrending using Empirical Mode Decomposition (EMD).

After deleting the 3 minutes of arterial pulse signal acquired during the occlusion phase, signals of the baseline and hyperemic phases were connected for analysis. The footpoint of each waveform was first marked, followed by the identification of the peak between two footpoints [23]. The amplitude of the waveform (*X*_*i*_, *i* = 1,2,…, 1000) was defined as the vertical distance between the peak and the nearest footpoint. The amplitudes *X*_1_, *X*_2_,…, *X*_379_ were defined as the baseline values of the arterial pulses, whereas the amplitudes *X*_380_, *X*_381_,…, *X*_1000_ were defined as the hyperemic phase values of the arterial pulses (see Figure 2(a)). The values of the amplitudes thus obtained were plotted versus time (see Figure 2(b)). Since the nonstationary nature of the curve would affect the accuracy of the multiscale entropy (MSE) calculation, the curve was detrended with empirical mode decomposition (EMD), as proposed by a previous study [24–26] (see Figure 2(c)). This process yielded 1,000 amplitude points {*X*_1_′, *X*_2_′, …, *X*_1000_′} for MSE analysis.

#### 2.4.1. Multiscale Entropy (MSE) Computation

MSE was calculated in accordance with the procedure reported by Costa et al. [16]. Given a 1-dimensional discrete time series, {*X*_1_′, *X*_2_′, …, *X*_1000_′}, consecutive coarse-grained time series {*y* ^(*τ*)^}, determined by the scale factor *τ*, can be constructed according to the equation

(2) $$\begin{array}{c} y_{j}^{\left(\tau \right)}=\frac{1}{\tau }\sum_{i=\left(j-1\right)\tau +1}^{j\tau }X_{i}^{\prime },\;1\leq j\leq \frac{1000}{\tau }, \end{array}$$

where *τ* denotes the scale factor and 1≦*j*≦1000/*τ*. In other words, coarse-grained time series for scale factor *τ* were acquired by taking the arithmetic average of *τ* neighboring original values without overlapping. The length of each coarse-grained time series is 1000/*τ*. For scale 1, the coarse-grained time series is just the original time series. Sample entropy (*S*_*E*_) [27] for each of the coarse-grained time series can be obtained and plotted against the scale factor, *τ*.

#### 2.4.2. Multiscale Entropy Index (MEI) Computation

The values of *S*_*E*_ were then obtained from a range of scale factors between 1 and 10 using the MSE data analysis method described above. The values of *S*_*E*_ between scale factors 1 and 5 were defined as small scale, whereas those between scale factors 6 and 10 were large scale [28, 29]. The sum of *S*_*E*_ values between scale factors 1 and 5 was defined as MEI_SS_, while the sum of *S*_*E*_ values between scale factors 6 and 10 was defined as MEI_LS_ [28, 29], see (3) below. By defining and calculating these two indices of multiscale entropy, the complexity of signals between different time scales can be assessed and quantified. Using these 2 indices, the present study attempted to evaluate the differences in signal complexity of the hyperemic responses elicited by temporary ischemia, an index of endothelial function, in different subject populations,

(3) $$\begin{array}{c} \text{ME}{\text{I}}_{\text{SS}}=\sum_{\tau =1}^{5}S_{E}\left(\tau \right),\\ \text{ME}{\text{I}}_{\text{LS}}=\sum_{\tau =6}^{10}S_{E}\left(\tau \right), \end{array}$$

where *S*_*E*_(*τ*) is the sample entropy for the respective scale factor.

### 2.5. Statistical Analysis

Average values were expressed as mean ± SD. Statistical Package for the Social Science (SPSS, version 14.0) was adopted. Independent sample *t*-test and Pearson's correlation were used for the determination of significance between different groups and assessment of correlations among different parameters, respectively. A *P* value <0.05 was considered statistically significant.

## 3. Results

**Figure 3:**
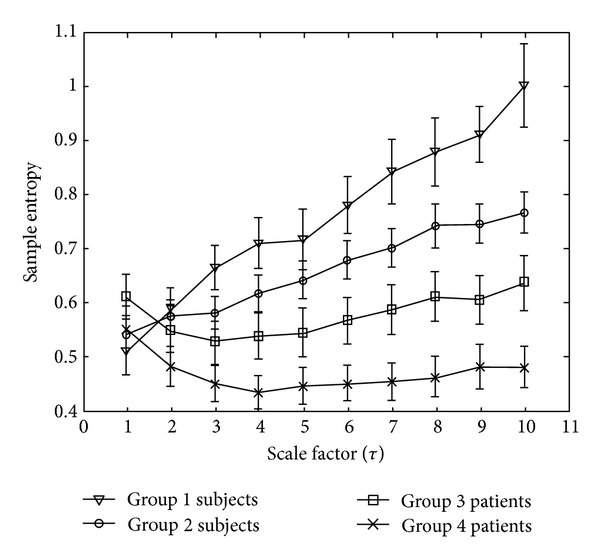
Changes in sample entropy (*S*_*E*_) with different scale factors in the four groups of subjects. Symbols represent the mean values of entropy for each group, and bars represent the standard error ($S_{E}=\text{SD}/\sqrt{n}$), where *n* is the number of subjects. Group 1: healthy young subjects; group 2: healthy middle-aged or elderly subjects; group 3: well-controlled diabetic subjects; group 4: poorly controlled diabetic subjects.

By recording serial changes in 1,000 arterial waveform amplitudes (between scale factor of 1 and 10) and analyzing their complexity (i.e., multiscale entropy) in 4 different subject populations of different ages and disease status, significant changes in *S*_*E*_ with different scale factors in the 4 different groups of subjects were noted (see Figure 3).

### 3.1. Changes in Sample Entropy, *S*_*E*_, with Scale Factor

The values of *S*_*E*_ decreased significantly from a scale factor of 6 onward in group 1 (healthy young subjects), group 2 (healthy middle-aged or elderly subjects), group 3 (well-controlled diabetics), and group 4 (poorly controlled diabetics) (see Figure 3). No significant difference in the values of *S*_*E*_ at lower scale factors (i.e., 1 to 5) was noted among the 4 groups.

### 3.2. Comparison between Healthy Young (Group 1) and Middle-Aged or Elderly (Group 2) Subjects

Remarkable differences were noted between healthy young (group 1) and middle-aged or elderly (group 2) subjects in terms of age, body height, HbA1c (*P* < 0.001), and serum HDL and LDL levels (*P* < 0.05; Table 1). Significant differences (*P* = 0.016) in DI were also noted between group 1 (201.57% ± 43.42%) and group 2 (164.88% ± 32.33%). No notable difference in MEI_SS_ was noted between the 2 groups (3.43 ± 1.23 versus 2.92 ± 0.89, *P* = 0.343); however, MEI_LS_ was significantly higher in group 1 than in group 2 (4.22 ± 1.41 versus 3.53 ± 0.99, resp., *P* = 0.025).

### 3.3. Comparison between Healthy Middle-Aged or Elderly (Group 2) and Well-Controlled Diabetic (Group 3) Subjects

**Figure 4:**
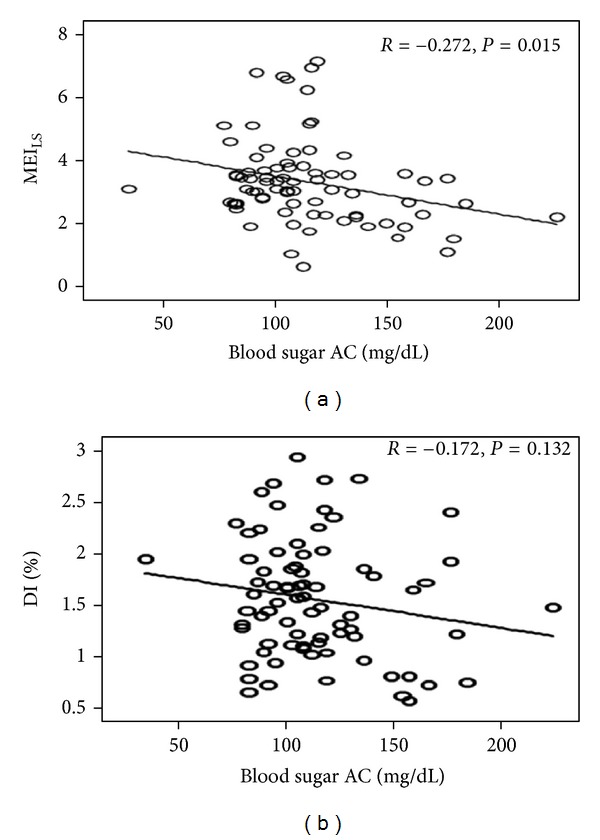
Correlations between (a) small-scale multiscale entropy index (MEI_LS_) and fasting blood sugar levels; (b) dilatation index (DI) and fasting blood sugar levels in healthy middle-aged or elderly (group 2) and well-controlled diabetic (group 3) subjects.

Table 1 summarizes the demographic, anthropometric, hemodynamic, and biochemical parameters, MEI, and DI between group 2 and group 3 (HbA1c <8%) subjects, showing notably advanced age, larger waist circumference, elevated HbA1c, and fasting blood sugar levels in the latter (*P* < 0.001). Body weight, body mass index, and systolic blood pressure in group 3 were significantly higher than that in group 2. On the other hand, serum LDL and HDL levels in group 3 were significantly lower than that in group 2 (*P* < 0.05). Multiscale entropy analysis revealed significantly higher MEI_LS_ in group 2 than that in group 3 (3.53 ± 0.99 versus 3.02 ± 1.48, resp., *P* = 0.037), whereas there was no notable difference in MEI_SS_ between the 2 groups (2.92 ± 0.89 versus 2.78 ± 1.27 for group 2 and group 3, resp., *P* = 0.452). In terms of DI, no remarkable difference was noted between group 2 and group 3 (164.88% ± 32.33%  versus  162.08% ± 35.34%, resp., *P* = 0.365). Moreover, a significant negative correlation was noted between MEI_LS_ and fasting blood sugar levels in the 2 groups (*R* = − 0.274, *P* = 0.015) (see Figure 4(a)), whereas no notable correlation could be found between DI and fasting blood sugar levels between these groups (*R* = −0.172, *P* = 0.132) (see Figure 4(b)).

### 3.4. Comparison between Well-Controlled (Group 3) and Poorly Controlled Diabetic (Group 4) Subjects

Although the subjects in group 3 (HbA1c <8%) were significantly older than those in group 4 (HbA1c >8%), the comparison between the 2 groups revealed significantly higher HbA1c, LDL, fasting blood sugar, and triglyceride levels in group 4 (Table 1). There was no significant difference in MEI_SS_ between group 3 and group 4 (2.78 ± 1.27 versus 2.37 ± 0.88, resp., *P* = 0.118); however, MEI_LS_ was remarkably higher in the well-controlled diabetic subjects (group 3) than that in the poorly controlled diabetic subjects (group 4) (3.02 ± 1.48 versus 2.34 ± 0.96, resp., *P* = 0.024). A notable difference in DI also existed between group 3 and group 4 (162.08% ± 35.34% versus 132.72% ± 36.57%, resp., *P* < 0.001).

### 3.5. Correlations of MEI_LS_ and DI with Anthropometric, Hemodynamic, and Biochemical Parameters

Attempts were made to correlate values of DI and MEI_LS_ from all subjects (*N* = 140) with their anthropometric, hemodynamic, and biochemical risk factors of CVD (Table 2). The results showed that DI was negatively correlated with waist circumference, body mass index, and HbA1c levels. On the other hand, while MEI_LS_ was negatively correlated with age, HbA1c, and fasting blood sugar levels, it was positively correlated with serum HDL levels.

## 4. Discussion

The human body consists of physiological systems of dynamical complexity involving a myriad of interactions and feedback mechanisms [17]. Recent studies [16–19], which placed strong emphasis on the quantification of dynamical complexity in healthy human subjects and those with cardiovascular diseases, have identified a reduction in dynamical complexity, defined by MSE, as a common characteristic of the aged and diseased subsets of the population. Previous applications of dynamical complexity analysis focused mainly on the study of *R*-*R* interval time series, in an attempt to investigate various cardiac diseases. For instance, compared with healthy subjects regardless of age, patients with congestive heart failure (CHF) have a higher *S*_*E*_ for scale 1 [16, 17]. In contrast, a lower *S*_*E*_ becomes apparent in subjects with CHF over scale 1. Analysis of *R*-*R* interval time series in normal subjects and in patients with ventricular arrhythmia and myocardial infarction revealed that *S*_*E*_ decreases with increasing age in both normal and diseased populations [24]. On the other hand, there is no significant difference in *S*_*E*_ between the healthy aged subjects and their counterparts with cardiac diseases. Moreover, healthy young subjects have the highest *S*_*E*_ at all scales compared with the aged and diseased groups [24].

The application of MSE in analyzing heart rate (HR) and systolic and diastolic blood pressure (BP) in 14 young patients with type 1 diabetes mellitus was first reported by Trunkvalterova et al. in 2008 [30]. MSE analysis of HR/BP signals showed a higher *S*_*E*_ value in the healthy subjects than that in the diabetic subjects on scale 3. Using age-matched healthy young subjects as normal controls, this study proposed that MSE is useful in detecting subtle vascular pathology in young diabetic subjects. However, the paradoxical result of MSE analysis on HR and diastolic BP in that study, which showed a higher *S*_*E*_ in diabetic patients compared with their healthy counterparts over scale 6, remains unexplained. The choice of a suitable physiological parameter is, therefore, essential in the successful application of MSE to the assessment of the degree of atherosclerosis and the effect of aging on vascular function.

Although the application of MSE using *R*-*R* interval time series in analyzing the dynamical complexity of cardiac diseases has been validated, reports on the use of MSE in assessing atherosclerotic change of blood vessels and the impact of age on the vascular system are rare.

Not only is endothelial dysfunction believed to precede microvascular changes of the cardiovascular system [1], it is also considered an indicator of atherosclerosis [1–4]. Previous studies have proposed a system of reactive RH-PAT, performed by the analysis of finger arterial pulse waves before and after applying pressure on the upper arm, in assessing vascular endothelial function. The popularity of its use, however, is restricted by the expensive equipment and the requirement of well-trained personnel for proper operation. The present study utilized APSS that we previously proposed to record the signals of arterial pulsations from the wrist before and after application of pressure on the upper arm [21]. After calculation of the DI, we attempted to assess vascular endothelial function by adopting MSE. We used it in calculating the dynamical complexity of the signals acquired from subjects belonging to different age groups and from subjects with different degrees of diabetic control, since diabetes and aging are both risk factors of atherosclerosis. In this manner, the two parameters of MEI_LS_ and MEI_LS_ were obtained and compared among the different groups.

Table 1 shows a notable difference in both MEI_LS_ and DI between healthy young (group 1) and middle-aged or elderly (group 2) subjects, whereas there was no significant difference in MEI_SS_ between the two groups. On the other hand, although DI did not differ between healthy middle-aged or elderly subjects (group 2) and well-controlled diabetic subjects (group 3), significant difference in MEI_LS_ existed between the 2 groups (Table 1). These results imply that MEI_LS_ can indicate subtle vascular changes even in well-controlled diabetic subjects, whose endothelial dysfunction is maintained at a relatively stable condition through lifestyle modification and medical control [7]. Further investigation revealed a negative correlation between fasting blood sugar levels and MEI_LS_, whereas the correlation between fasting blood sugar levels and DI failed to reach statistical significance. In term of HbA1c levels, a better correlation was noted with MEI_LS_ (*P* < 0.001) than with DI (*P* = 0.013) (Table 2). Taken together, the findings suggest that MEI_LS_ may serve as a better indicator of subtle diabetes-associated vascular endothelial dysfunction and sugar control than DI, indicating the possible use of MEI_LS_ as a sensitive indicator of vascular endothelial dysfunction that allows early therapeutic intervention.

When DI and MEI_LS_ were compared in terms of their correlations with the risk factors of CVD (Table 2), significant correlations were noted between DI and waist circumference (*R* = −0.193, *P* = 0.043), body mass index (*R* = −0.162, *P* = 0.043), and HbA1c (*R* = − 0.223, *P* = 0.013), whereas significant correlations existed between MEI_LS_ and age (*R* = −0.223, *P* = 0.012), HbA1c (*R* = −0.375, *P* < 0.001), serum HDL (*R* = 0.240, *P* = 0.010), and fasting blood sugar levels (*R* = −0.344, *P* < 0.001). The results further suggest that MEI_LS_ may be a more sensitive indicator of endothelial dysfunction associated with aging and diabetes than DI. The superiority of MEI_LS_ over DI may be due to the fact that the latter utilizes two segments of representative 1-minute signals acquired before and after vascular occlusion, whereas the former analyzes all 14-minute signals from both the baseline and hyperemic phases using the MSE technique.

This study has unavoidable limitations. First, since the computation of MEI requires time-consuming detrending of signals and extensive MSE analysis, immediate information cannot be provided for the examinees. This problem can probably be solved by the development of appropriate software for data analysis. Second, the current study only recruited a relatively small number of subjects and focused on only a single disease. Further investigation is warranted to include a larger number of patients with diseases related to endothelial dysfunction, including stroke, angina, limb ischemia, and erectile dysfunction. Finally, the requirement for an occlusion pressure of up to 200 mmHg over the upper arm for 3 minutes may not be tolerated by some study subjects. This was the situation for 3 of our diabetic patients, who were subsequently excluded from the present study.

## 5. Conclusion

Using the method of MSE for nonlinear dynamical analysis of arterial pulse signals from the wrist, this study successfully detected subtle differences in dynamical complexity of the acquired signals from the young, the middle-aged or elderly, well-controlled, and poorly controlled diabetic subjects using the novel parameter MEI.

## Figures and Tables

**Table 1:** Comparison of demographic, anthropometric, hemodynamic and biochemical parameters, MEI, and DI between healthy young subjects (Group 1), healthy middle-aged or elderly subjects (Group 2), well-controlled diabetic subjects (Group 3) and poorly controlled diabetic subjects (Group 4).

Parameter	Group 1	Group 2	Group 3	Group 4
*N*	30	40	40	30
Age (years)	24.87 ± 2.69	56.59 ± 8.75**	64.98 ± 9.26^++^	60.03 ± 8.24^*ε*^
Body height (cm)	172.63 ± 6.86	161.93 ± 7.44**	160.55 ± 8.56	163.26 ± 7.16
Body weight (kg)	68.12 ± 10.99	63.31 ± 10.70	68.09 ± 10.28^+^	71.41 ± 11.93
Waist circumference (cm)	80.97 ± 9.55	82.11 ± 9.92	93.13 ± 9.37^++^	93.06 ± 11.62
BMI (kg/m^2^)	22.79 ± 3.06	24.11 ± 3.59	26.40 ± 3.39^+^	26.98 ± 5.30
SBP (mmHg)	116.18 ± 12.31	118.11 ± 15.19	128.34 ± 17.02^+^	126.83 ± 17.66
DBP (mm Hg)	71.94 ± 6.18	73.94 ± 10.49	75.04 ± 10.14	74.72 ± 11.19
HbA_1c_ (%)	5.49 ± 0.25	5.67 ± 0.31**	6.79 ± 0.60^++^	9.85 ± 1.81^*εε*^
HDL (mg/dL)	44.81 ± 5.60	52.94 ± 20.64*	42.78 ± 16.26^+^	43.39 ± 14.65
LDL (mg/dL)	97.0 ± 26.83	122.48 ± 26.78*	99.33 ± 25.17^++^	117.93 ± 36.23^*ε*^
Fasting blood sugar (mg/dL)	92.69 ± 3.19	97.70 ± 15.76	128.06 ± 28.77^++^	166.96 ± 59.07^*ε*^
Triglyceride (mg/dL)	89.31 ± 60.14	105.09 ± 51.06	110.29 ± 41.71	161.85 ± 53.72^*ε*^
Creatinine (mg/dL)	0.92 ± 0.12	0.79 ± 0.22*	0.93 ± 0.37	1.24 ± 1.17
Microalbumin (mg/dL)	0.72 ± 0.56	0.64 ± 0.66	16.99 ± 57.99	71.68 ± 222.41
MEI_SS_	3.43 ± 1.23	2.92 ± 0.89	2.78 ± 1.27	2.37 ± 0.88
MEI_LS_	4.22 ± 1.41	3.53 ± 0.99*	3.02 ± 1.48^+^	2.34 ± 0.96^*ε*^
DI (%)	201.57 ± 43.42	164.88 ± 32.33*	162.08 ± 35.34	132.72 ± 36.57^*εε*^

Value are expressed as mean ± SD. BMI: body mass index; SBP: systolic blood pressure; DBP: diastolic blood pressure; HbA_1c_: glycosylated hemoglobin; HDL: high density lipoprotein; LDL: low density lipoprotein; MEI_SS_: Multiscale Entropy Index with Small Scale; MEI_LS_: Multiscale Entropy Index with Large Scale; DI: Dilatation Index. *P < 0.05: Group 1 versus Group 2, ^+^ *P* < 0.05: Group 2 versus Group 3, ^*ε*^ *P* < 0.05: Group 3 versus Group 4. **P < 0.001: Group 1 versus Group 2, ^++^ *P* < 0.001: Group 2 versus Group 3, ^*εε*^ *P* < 0.001: Group 3 versus Group 4.

**Table 2:** Correlations of MEI_LS_ and DI with anthropometric, hemodynamic, and biochemical parameters.

Parameter	DI (*N* = 140)	MEI_LS_ (*N* = 140)
Age (years)	*R* = − 0.168, *P* = 0.062	*R* = − 0.223, *P* = 0.012
Body height (cm)	*R* = 0.113, *P* = 0.144	*R* = − 0.063, *P* = 0.440
Body weight (kg)	*R* = − 0.078, *P* = 0.423	*R* = − 0.127, *P* = 0.147
Waist circumference (cm)	*R* = − 0.193, *P* = 0.043	*R* = − 0.143, *P* = 0.117
BMI (kg/m^2^)	*R* = − 0.162, *P* = 0.043	*R* = − 0.092, *P* = 0.309
SBP (mmHg)	*R* = − 0.183, *P* = 0.054	*R* = − 0.031, *P* = 0.735
DBP (mmHg)	*R* = − 0.124, *P* = 0.195	*R* = 0.007, *P* = 0.937
HbA_1c_ (%)	*R* = − 0.223, *P* = 0.013	*R* = − 0.375, *P* < 0.001
HDL (mg/dL)	*R* = 0.034, *P* = 0.730	*R* = 0.240, *P* = 0.010
LDL (mg/dL)	*R* = − 0.070, *P* = 0.478	*R* = − 0.025, *P* = 0.791
Fasting blood sugar (mg/dL)	*R* = − 0.169, *P* = 0.074	*R* = − 0.344, *P* < 0.001
Triglyceride (mg/dL)	*R* = − 0.165, *P* = 0.091	*R* = − 0.158, *P* = 0.088

BMI: body mass index; SBP: systolic blood pressure; DBP: diastolic blood pressure; HbA_1c_: glycosylated hemoglobin; HDL: high density lipoprotein; LDL: low density lipoprotein.
